# Medical Department University of Michigan and the Peninsular Journal

**Published:** 1855-09

**Authors:** 


*Medical Department University of Michigan and the Peninsular Journal.*

In the May No. of this journal, we inserted a paragraph, simply pointing out what we deemed an unwarrantable assumption of *superiority* in the system of instruction given in the Medical Department of the University of Michigan, as set forth by the editor of the Peninsular Journal, Prof. A. B. Palmer. If our readers will turn to that number of the Journal, they will see that neither in the paragraph alluded to, nor in the whole article of which it was a part, did we say one unkind word in regard to the Ann Arbor school, or make the least comparison between it and any other school of medicine. And yet it has served to call out from Dr. Palmer *seven* pages in the July number of the Peninsular Journal. The first page and a half of this is made up of comments on confidence in the intelligence of the people of Michigan; another page is devoted to the benevolent work of informing his readers who the senior editor of this Journal is, what he has done heretofore, and what are his present personal relations to medical schools, reforms, etc.; while the remaining four pages and a half are devoted more particularly to the subject under consideration.

With the first part of Dr. Palmer's article we have nothing to do. The second, if it has any object or purpose, it is to lessen the confidence of his readers in my own personal and professional character,—a motive unworthy of an honorable rival and much more of a personal friend. We shall therefore pass it also in silence, leaving the author to gather all the gratification from it which his conscience will allow. The third division of his article however, shall receive all the attention its merits demand. And for this purpose our readers will pardon us for taking the time and space to call up a few items in the history of the past.

In the March or April number of the Peninsular Journal, its senior editor, who is also a professor in the Medical Department of the University of Michigan, devoted several pages to a notice of Dr. Cabell's Report on Medical Education, prepared for the Annual Meeting of the American Medical Association held in May, 1853. In doing so, he brought prominently before his readers such parts of the report as would appear most favorable to the course pursued by the medical faculty of the school of which he was a member. Of this we made no complaint; but he went further, and expressed his pride in the following language: "We are *proud* that the Medical Department of the University of Michigan *is so far in advance* of almost every other school in the country, in the cause of reform herein so ably pointed out."

This, if it meant anything, most clearly meant to convey the impression to his readers, that in its course of instruction and standard of requirements, the Medical Department of the University of Michigan was "far in advance" of all the other medical schools. It was this that we characterized as an "arrogant flourish" and a "baseless pretension." This was our plain, direct accusation, and the editor of the Peninsular Journal, instead of adducing proof that the accusation was unfounded, declares himself "pained" and surprised, and then dodges the whole question by getting up a most unfair and partial comparison between the requirements of the Michigan University and Rush Medical College.

Now we propose, first, to prove our original accusation to be *literally true;* and second, to show that in Dr. Palmer's pretended comparison of the two schools, he has been most unfair and unmanly. That our readers may be able to judge for themselves whether the Michigan school "*is so far in advance of almost every other school,*" in the cause of reform so ably pointed out by Dr. Cabell, or not, we will quote a summary of his views in his own language, as follows:

"Your Committee beg leave, in conclusion, to submit to your consideration the following resolutions, as a summary of the principle views embodied in the foregoing Report.

"1. *Resolved*, That the views and recommendations heretofore expressed by this Association, respecting the importance of establishing a uniform standard of preliminary education, of extending the term of lectures, and especially of greatly elevating the standard of professional attainments requisite to graduation, be hereby reaffirmed.

"2. *Resolved*, That this Association approves and recommends the practice of daily examinations by each professor, as essential for securing 'that active, practical discipline' of the mind which is one of the most important ends of collegiate instruction; and believes, not only that such a system might be easily put into operation, under an extension of the term of lectures, but that the whole ground-work of elementary medical instruction might be most advantageously assigned to the schools which may adopt that system, as a substitute for the very faulty one of private office instruction now in common use.

"3. *Resolved*, That this Association cordially approves of the establishment of "private schools" duly organized for giving that species of instruction which consists 'in demonstrations' and other practical exercises 'on the part of the student, instead of the instructor, but still under his direction and superintendence,' embracing the whole circle of clinical observation and practice, the use of the microscope, chemical manipulations, and the performance of surgical operations on the dead body; and it would earnestly recommend such institutions to the patronage of those graduates who did not enjoy similar advantages during the period of their collegiate pupilage.

"4 *Resolved*, That those medical colleges whose curriculum does not now include full courses of lectures on physiology and medical jurisprudence, be earnestly invited to make immediate provision for supplying the deficiency, and to require the professor of physiology to make an exposition of the outlines of comparative anatomy, to such extent, at least, as may be necessary to enable the student to appreciate the force of the evidence upon which the modern doctrines of physiology mainly rest."

Here we have a "summary" of Dr. Cabell's views in his own words; but to comprehend their full bearing we must go back to those previous recommendations of the Association, which Dr. Cabell in his first resolution proposes to have *re-affirmed.*

These may be found in the volumes of transactions of the Association for the years 1847-49-53; and are as follows, viz.:

" *Resolved*, 1. That it be recommended to all the colleges to extend the period employed in lecturing, from four to six months.

"2. That no student shall become a candidate for the degree of M.D., unless he shall have devoted *three entire years to the study of medicine*, including the time allotted to attendance on lectures.

"3. That the candidate shall have attended two full courses of lectures, that he shall be twenty-one years of age, and in all cases shall produce the certificate of his preceptor, to prove when he commenced his studies.

"4. That the certificate of no preceptor shall be received who is avowedly and notoriously an irregular practitioner, whether he shall possess the degree of M.D or not.

"5. That the several branches of medical education already named in the body of this report, be taught in all the colleges, and that the *number of professors be increased to seven.*

"6. That it be required of candidates that they *shall have steadily devoted three months to dissections.*

"7. That it is incumbent upon preceptors to avail themselves of every opportunity to impart clinical instruction to their pupils; and upon *medical colleges to require candidates for graduation to show that they have attended on hospital practice for one session*, whenever it can be accomplished, for the advancement of the same end."

"*Resolved*, That the Association *does not sanction or recognize* '*college clinics*' as *substitutes* for *hospital clinical instruction;* and *that the medical colleges be again advised to insist*, in all instances where it was practicable, on the regular attendance of their pupils, *during a period of six months, upon the treatment of patients in a properly conducted hospital*, or other suitable institution devoted to the reception and cure of the sick."

"*Resolved*, That in the opinion of this Association, a familiar knowledge of the elements of medical science should precede clinical instruction.

"*Resolved*, That in order to accomplish the latter, the hospitals, when they shall be elevated to the rank of schools of practice, and the intelligent private preceptor, are the most effectual instrumentalities to be employed."

It is proper here to remark that neither the report of Dr. Cabell, nor the "summary" embodied in the resolutions quoted from him, were ever read to or in anywise adopted by the Association; and they consequently stand on the pages of the transactions merely as the sentiments of their author. All the other resolutions quoted, however, were fully presented and *adopted* by that body. Now, let us see wherein the Michigan University is "*so far in advance*" of other respectable schools, in regard to the requirements and recommendations above fully and fairly quoted.

On a careful examination, the reader will find the only recommendations contained in the resolutions of Dr. Cabell, and not specifically contained in the other resolutions previously adopted by the Association, are first, "the practice of daily *examinations* by each professor;" and secondly, the establishment of "Private Schools" for certain specified purposes.

In regard to the first of these—the daily examinations by each professor—Dr. Palmer, with quite as little regard for truth as modesty, makes the following assertion, viz.: "This is only done in a partial and limited manner in Rush Medical College, and by only a part of the professors." Now we happen to know that *every* professor in the faculty of Rush Medical College, and also in a large number of other colleges in this country, *does* make it a rule to practice daily examinations of the classes in their several institutions. And hence, if his assertion to the contrary is not another "*arrogant flourish*," it certainly is not remarkable for its

*modesty ;* and until we have some other proof of their superior *advancement* in this particular than his interested assertion, the profession will know how much importance to attach to it. The other recommendation in regard to the establishment of private schools, is conceded to have no bearing on the present controversy. Having disposed of wnat is peculiar in the resolutions of Dr. Cabell, let us inquire concerning the *advance* or superiority of the Michigan school in the adoption of all the other measures specifically recommended by the American Medical Association itself. They are all essentially included, so far as Medical Colleges are concerned, in the seven resolves quoted and numbered above. And that our readers may again judge for themselves we will again quote the exact requirements of the Michigan school, not as *amplified* by Dr. Palmer in his editorial, but as published in their recent annual announcement for 1855.

They are as follows, viz.: "Each candidate for admission must be provided with satisfactory evidence of good moral character; and, if a candidate for graduation, also of such literary attainments as have been recommended by the American Medical Association. To be admitted to the degree of Doctor of Medicine, the student must exhibit evidence of having pursued the study of medicine and surgery for the term of three years with some respectable practitioner of medicine, (including lecture terms), must have attended two full courses of lectures, the last of which must have been in the college of Medicine and Surgery of the University of Michigan, and the previous one in this or some other respectable Medical Institution; must have been engaged in the study of practical Anatomy; must be twenty-one years of age, must have submitted to the Faculty a Thesis, composed and written by himself, on some medical topic, and have passed an examination at the close of the term, satisfactory to the Faculty. To encourage a higher grade of preliminary acquirement, an allowance of *one year* from the term of study is made in favor ot graduates of the College of Science and Arts, and of other respectable Literary Colleges. Four years of respectable practice *is received in lieu* of one course of lectures. Each candidate for graduation must so announce himself at the commencement of his second course, and must be examined in anatomy, physiology, materia medica, and chemistry. He is also required to write and defend a medical essay once in two weeks."

Here, instead of requiring the devotion of "three entire years to the study of medicine," as recommended in the second resolve quoted above, is a provision for conferring the degree of Doctor of Medicine *after only two years study*; instead of requiring in all cases attendance on "two full courses of lectures," as recommended in the third resolve, we heve provision for admitting certain candidates after attendance on only *one course*; instead of having *seven* acting Professors in their Faculty, as recommended in the fifth resolve, they have only *six*; instead of requiring their candidates to devote "three months to dissections," as recommended in the sixth resolve, they simply say "he must have been engaged in the study of Practical Anatomy;" and finally, instead of requiring attendance "on *hospital practice*, for *one session*, as recommended in the *seventh* resolve, they are either entirely silent on the subject or graciously tell their classes, that they must depend for this important part of medical instruction on the practitioners of the *rural* districts. Thus we find by direct comparison, that the Medical Department of the University of Michigan, either completely or partially fails to comply with *five* out of the *seven* formally established recommendations of the American Medical Association; recommendations too, which Dr. Cabell, himself wished to have "*reaffirmed.*"

And yet with all these glaring delinqencies, the Faculty of that school have lost no opportunity to impress upon the profession the idea that they had fully adopted the course of instruction and the requisitions recommended by the Association. Thus in their annual announcement for the session of 1852-3, they declare that; "The *peculiar* position of the College of Medicine and Surgery of the University of Michigan, is such that it has been *enabled fully to comply with the demands of the profession*," *&c.*

The same impression is sought to be made by Dr. Z. Pitcher, an emeritus professor in the same school, in his report on Medical education, read to the American Medical Association, in May 1853, in which he uses the following language: "By the medical faculty of the University, all candidates for the degree of M. D., *are rigidly required to comply with the conditions of the National Medical Association*, except attendance on hospital cliniques," &c. And at the same meeting of the Association, Dr. Palmer, himself *seconded* ,with his accustomed *modesty*, and strongly urged the adoption of a resolution specially endorsing and commending the medical department of their University. But finding a continuance of the discussion likely to bring before the association some of their important deficiencies, the resolution was withdrawn. They were not content, however, to stop here. For at the last annual meeting of the Association the same Dr. Palmer, procured the introduction by Dr. Atlee, of Pa., of resolutions condemning any attempt to introduce a chair of Homœopathy into regular schools of Medicine. The resolutions were right enough and were adopted. But on them Dr. Palmer made a speech in defence of his favorite University, in which, as reported by himself, we find the following language, viz. : "an institution which had sought to comply with the recommendations of this body." And again : "The founders of the medical department of the University of Michigan, sought in its organization to follow your directions," &c Now let the reader compare these repeated claims to a compliance with the important recommendations of the National Association, and these well studied efforts to procure from that body some special endorsement, with their actual *requirements*, as quoted directly from their last annual announcement, and then judge whether our charge of "arrogant flourishes" and "baseless pretensions," was well founded or not. It is true that they have adopted a *long* lecture term. But so has one of the Medical Colleges in Virginia ; so has the Medical department of the University of Pennsylvania ; so has the College of Physicians and Surgeons of New-York, and some others. And hence they have no valid claim to being "*far in advance* of almost every other school," in this respect. All they have left in the whole list of their requisition, on which they can rest a claim of *advancement*, may be seen in the first and last paragraphs of their requirements, as quoted above. The first declares that candidates for graduation "must be provided with satisfactory evidence of such literary attainments as have been recommended by the American Medical Association;" and the last that they "must write and defend a Medical Essay once in two weeks." This latter, as explained in the article of Dr. Palmer, seems to be a kind of primary school exercise in the art of writing *compositions*.

When we put along side of these imperfect requirements in regard to literary attainments, their provision for deducting *one third* from the usual period of Medical study; for dispensing in some cases with *one course* of lectures; and their failure to exact attendance, at least, one term on practical dissections and hospital clinical instruction, we are forcibly reminded of a certain class in ancient times, who "paid tithes of mint and cummin and anise," but "neglected the weightier matters of the law," &c. Here we would willingly leave this subject, had not Dr. Palmer in his effort to escape from the charge of having made "baseless pretensions," instituted a most unfair and uncalled for comparison between the requisitions of the Michigan University and those of Rush Medical College. In making this comparison he is careful to state fully, and even to *amplify*, all the requirements of the school with which he is connected at Ann Arbor, enumerating the *certificates* of term of study, preliminary education, moral character, theses, examinations, &c., &c.; and in immediate contrast with them, instead of quoting the requisitions of Rush Medical College, as published in its annual announcement and rigidly enforced by its faculty; he places nothing but the following extraordinary paragraph, viz.: "That Institution (Rush Med. College) admits to the graduating class all who have studied the usual time, attended two courses of lectures of sixteen weeks, and who writes and hands in a thesis, which is seldom, perhaps, read—never by the student before the faculty or class" We called this an extraordinary paragraph, and it certainly is so, for it violates the rules of *grammar*—sets at nought the common courtesies of life—and in the position in which it was placed by its author, it conveys the most glaring falsehoods.

The broad assertion here made, that the Faculty of Rush Medical College admits to her graduating class all who have simply studied the usual time, attended two courses of lectures, and have written and handed in a thesis, leaves the reader to infer, as a matter of course, that no attention is paid to evidence or certificates of age, moral character, term of study, place of previous attendance on lectures, &c., &c.,—an inference which Dr. Palmer knows to be utterly false. And yet, if he did not *wish* such an erroneous inference to be drawn by his readers, why did he not quote our requirements in full, as he did those of the school with which he was himself connected. Had he done so fairly and honorably, his readers would have seen, that, instead of failing to comply with *five* out of the *seven* specific recommodations of the American Medical Association in regard to Medical Colleges, Rush Medical College falls short in only *two*, namely, the length of the lecture term and the permission of four years practice to take the place of one course of lectures. If by the foregoing observations our friend, the editor of the *Peninsular Journal*, finds himself and the school with which he is connected, placed before the profession in no enviable light, he may blame no one but himself and his colleagues. Placed in a school richly endowed by the State, and paid fixed salaries wholly independent of their classes; the profession had a right to expect that they would take a high and firm stand in the cause of Medical Education; demanding in *all cases* the full period of Medical Study; attendance on at least *two* courses of lectures; and a full compliance with the recommendations of the profession in regard to literary attainments, personal attention to dissections, and to clinical instruction. How they have answered these just expectations the reader can judge from the facts and records already adduced.

